# A Randomized Controlled Trial With an Internal Pilot of a Co‐Designed App Targeting Upstream Suicide‐Related Risk and Protective Factors Among International Students

**DOI:** 10.1111/sltb.70125

**Published:** 2026-07-02

**Authors:** Samuel McKay, Christina Ng, Jennifer Nicholas, Vivienne Browne, Gina Chinnery, Isabella Choi, Bailey Nation‐Ingle, Kristal Allison, Ella Perlow, Michelle Lamblin, Elise Carrotte, Ellie Brown, Gregory Armstrong, Jocelyn I. Meza, Madhavan Mani, Jo Robinson

**Affiliations:** ^1^ Orygen Parkville Australia; ^2^ School of Psychological Sciences Monash University Melbourne Australia; ^3^ Centre for Youth Mental Health The University of Melbourne Melbourne Australia; ^4^ Sydney Medical School, Faculty of Medicine and Health The University of Sydney Sydney Australia; ^5^ Victorian Department of Health, Victorian Government Suicide Prevention and Response Office Melbourne Australia; ^6^ Australian Research Council's Centre of Excellence for Children and Families Over the Life Course, Australian Research Council Melbourne Institute Melbourne Australia; ^7^ Centre for Mental Health and Community Wellbeing, Melbourne School of Population and Global Health The University of Melbourne Melbourne Australia; ^8^ Department of Psychiatry and Biobehavioral Sciences University of California Los Angeles California USA; ^9^ Thriver Consulting Moggill Queensland Australia

**Keywords:** emotion regulation, foreign students, help‐seeking, mobile health, self‐guided intervention, transdiagnostic mechanisms

## Abstract

**Background:**

International students frequently report suicidal thoughts, yet often do not access support. Bud is a co‐designed, self‐guided mobile app developed as an upstream suicide‐prevention intervention targeting transdiagnostic mechanisms related to suicide risk.

**Aim:**

To evaluate the effectiveness, acceptability, engagement, and safety of Bud compared with an active and structured psychoeducational comparator.

**Materials and Methods:**

A two‐arm, parallel‐group randomized controlled trial was conducted with international students in Australia. Participants were randomized 1:1 to Bud (*n* = 156) or psychoeducational fact sheets (*n* = 156), completing assessments at baseline, 2 weeks and 4 weeks. The primary outcome was psychological distress (K10). Secondary outcomes included help‐seeking intentions, interpersonal risk processes, emotion regulation, suicidal ideation, and mental health literacy. Analyses used intention‐to‐treat mixed‐effects models.

**Results:**

Contrary to the hypotheses, psychological distress decreased from baseline to 4 weeks (Bud: −2.23; Comparator: −2.15), with no group × time interaction (*F*(2,342.45) = 0.43, *p* = 0.652); emotion regulation and suicidal ideation showed similar patterns across groups, and other secondary outcomes remained stable across groups. As expected, Bud was rated as enjoyable, worthwhile, and usable. Engagement was moderate (median 10 activities), and no iatrogenic effects occurred.

**Conclusion:**

Bud was feasible, acceptable, and safe but did not outperform an active psychoeducational comparator over 4 weeks.

**Trial Registration:** ClinicalTrials.gov identifier: ACTRN12625000584437.

**Abbreviations**
ACT: acceptance and commitment therapy
CBT: cognitive behavioral therapy
DBT: dialectical behavior therapy
DERS‐SF: difficulties in emotion regulation scale, short form
GHSQ: General Help‐Seeking Questionnaire
INQ‐15: Interpersonal Needs Questionnaire (15 items)
K10: Kessler Psychological Distress Scale (10 items)
MHLS‐9: Mental Health Literacy Scale, 9‐item version
OECD: Organization for Economic Co‐operation and Development
RAG: Red–Amber–Green (feasibility threshold framework)
RCT: Randomized Controlled Trial
REDCap: Research Electronic Data Capture
SIMR: Simulation‐Based Power Analysis for Mixed Models (R package)
uMARS: User Version of the Mobile App Rating Scale
YSIS‐3: Youth Suicidal Ideation Screen, 3‐item version

## 1 Introduction

Suicide is a leading cause of death among young people, with high rates reported among students in higher education (Mortier et al. 2018). International students comprise a large portion of students in many countries (OECD 2023) and report similar past‐year rates of suicidal ideation and self‐harm to domestic students, but higher rates of suicide attempts (Veresova et al. 2024). Evidence suggests that suicidal thoughts and behavior among international students are associated with academic pressure, acculturative stress, stigma, and limited access to familiar support networks (Veresova et al. 2024; Maharaj et al. 2025).

Despite growing awareness of these risks, suicide prevention efforts for international students within tertiary education remain limited and continue to rely on conventional approaches such as clinical treatment and broadly targeted mental health information (McKay et al. 2023). These models are poorly matched to the realities of international student populations (McKay and Meza 2024). International students, including those who die by suicide, are less likely to engage with formal mental health services due to stigma, misinformation about potential visa loss, low mental health literacy, and high treatment costs (McGregor 2023; Jamieson 2021; Clough et al. 2019; Zhou et al. 2022). Among those who do seek help, services are often difficult to access at the point of need, insufficiently adapted to students' cultural contexts, or misaligned with their expectations, resulting in poor uptake and early disengagement (Sanci et al. 2022; Bartholomew et al. 2022). Collectively, these limitations point to the need for suicide‐prevention strategies that are co‐developed with international students themselves and delivered in ways that are timely, contextually relevant, and culturally responsive (McKay and Meza 2024).

Digital interventions represent a promising avenue to address limitations with existing support models because they can be accessed privately, delivered at scale, including to those who do not have access to universal healthcare, and tailored to the needs of culturally diverse student groups (McKay and Meza 2024; Choi et al. 2023). Existing digital mental health interventions and psychoeducation programs for university students have shown benefits for mental health literacy, psychological distress, and, in some cases, suicidal ideation (Burr et al. 2025; Garrido et al. 2019; Lattie et al. 2019; Torok et al. 2020). Evidence also suggests that such interventions are generally acceptable to university students (Burr et al. 2025; Madrid‐Cagigal et al. 2025; Taylor et al. 2024). However, most digital programs have been developed for general student populations (King et al. 2022; Torok et al. 2022), and no randomized controlled trials have evaluated a digital suicide‐prevention intervention co‐designed specifically with international students (McKay et al. 2023).

Bud is a self‐guided mobile app developed through an extensive co‐design process with international students to address proximal precursors of suicide risk in culturally responsive ways. It is designed as a community‐level universal suicide‐prevention program focused on strengthening protective factors and reducing key psychological drivers of suicide risk, rather than providing clinical treatment. Bud adopts a transdiagnostic approach targeting mechanisms known to contribute to psychological distress and increase risk for suicidal thoughts and behaviors, including emotion regulation difficulties, perceived burdensomeness, low sense of belonging, negative thinking patterns, and broader stress‐related coping deficits (Dalgleish et al. 2020; Batterham et al. 2021). These mechanisms are targeted through brief, evidence‐based strategies embedded within the app, including psychological tools, peer narratives that normalize distress and encourage help‐seeking, and a brief suicide prevention skills course (McKay et al. 2025). Together, these components aim to influence modifiable psychological and interpersonal pathways linked to suicide risk (Veresova et al. 2024; Burr et al. 2025; McKay et al. 2025; Van Orden et al. 2010).

Consistent with this approach, psychological distress was selected as the primary outcome due to its high prevalence among international students (Skromanis et al. 2018; Xiong et al. 2025), its robust association with suicidality (Chamberlain et al. 2009), and its responsiveness to low‐intensity digital interventions (Dingwall et al. 2023; Dear et al. 2018). Secondary outcomes were selected to capture key suicide‐related mechanisms targeted by the intervention, including help‐seeking intentions, perceived burdensomeness, belongingness, emotion regulation, suicidal ideation, and mental health literacy (McKay et al. 2025). These constructs are well‐established correlates and predictors of suicidal thoughts and behaviors (Veresova et al. 2024; Richardson et al. 2024), and represent modifiable pathways through which intervention effects may reduce suicide risk over time (Burr et al. 2025; Torok et al. 2020).

This study reports findings from a parallel‐group randomized controlled trial which aimed to evaluate the effectiveness, acceptability, engagement, and safety of Bud compared with an active psychoeducational fact sheet condition. The primary outcome was psychological distress, with secondary outcomes including help‐seeking intentions, perceived burdensomeness, sense of belonging, emotion regulation, suicidal ideation, and mental health literacy. Consistent with its positioning as a universal community‐level, upstream suicide‐prevention intervention, the trial was not designed to target only individuals with current suicidal ideation; instead, it aimed to reduce broader psychological distress and related risk processes that precede and contribute to suicidal thoughts and behaviors. We hypothesized that:

### 1.1 Primary Hypothesis

*Participants using Bud would show greater reductions in psychological distress at post‐intervention compared with the comparator group*.

### 1.2 Secondary Hypotheses

*Bud users would show greater increases in help‐seeking intentions*.

*Bud users would show greater reductions in perceived burdensomeness and thwarted belongingness*.

*Bud users would show greater improvements in emotion regulation*.

*Bud users would show greater increases in mental health literacy*.

*Bud users would show greater reductions in suicidal ideation*.

*Bud would be acceptable to international students, including positive evaluations of engagement, functionality, esthetics, information quality, and overall quality*.

*Bud users would demonstrate high engagement, reflected in app‐usage metrics and perceived helpfulness of activities*.

*Bud would be safe, with no evidence of iatrogenic effects such as increased distress or suicidal ideation*.

## 2 Materials and Methods

### 2.1 Trial Design

The study used an open‐label, parallel‐group randomized controlled design comparing the Bud app with psychoeducational fact sheets. The trial included an internal pilot phase using the prespecified Red–Amber–Green (RAG) framework (Avery et al. 2017) to assess feasibility across recruitment (≥ 50% of target within 6 months), retention (≥ 60% completing follow‐up surveys), and engagement (≥ 50% using the app at least three times). The trial was prospectively registered with the Australian New Zealand Clinical Trials Registry (Registration number: ACTRN12625000584437), and a published trial protocol is available (McKay et al. 2025). Ethical approval was obtained from the University of Melbourne Human Research Ethics Committee (ID: 32102). All participants provided informed consent.

### 2.2 Participants

Participants were a community sample recruited online through university mailing lists, institutional communication channels, and social media pages serving international students in Australia. Eligible participants were international students enrolled at an Australian tertiary education institution and residing onshore, aged 18 years or older, able to read and speak English, and who had access to an Android or iOS smartphone. Suicidal ideation was not required for participation, as the intervention was designed as a community‐level, upstream suicide prevention program. There were no predefined exclusion criteria.

### 2.3 Procedures

Recruitment occurred between July and September 2025. Assessments were administered at baseline (T1), 2 weeks post‐baseline (T2), and 4 weeks post‐baseline (T3). Follow‐up assessments were completed by October 2025. The trial was conducted remotely, with participants completing all study procedures online, including consent and baseline and follow‐up assessments via REDCap. Randomization occurred automatically within REDCap, using an automatic randomization module, when participants progressed to the final section of the baseline survey (approximately 90% completion), using a stratified block randomization procedure; no study staff were involved in assignment. Stratification was based on baseline suicidal ideation (Youth Suicide Ideation Screen—3; YSIS‐3 (Hetrick et al. 2021)) across three predefined strata (0–3, 4–6, 7+), using an allocation ratio of 1:1 with a block size of six to ensure balanced allocation. The randomization sequence was generated in R by a researcher independent of participant management, ensuring allocation concealment until the point of assignment. Participants enrolled themselves via REDCap. Because of the nature of the intervention materials, participants were not blinded to group allocation.

Participants allocated to the Bud condition were directed to download the app from the iOS or Google Play store to complete the intervention on their personal smartphones, while those in the fact sheets group accessed their materials directly within REDCap. Participants were paid AUD $15 per survey completed as compensation for their time.

The statistician was blinded to group allocation during model scripting but could see condition labels during final analysis. Although the study incorporated an internal pilot using feasibility thresholds, no interim analyses of outcomes or statistical stopping guidelines were specified. The study was coordinated by researchers at Orygen and the Centre for Youth Mental Health at the University of Melbourne.

### 2.4 Intervention and Comparator

Participants were assigned to one of two conditions for 4 weeks: the Bud app (intervention) or psychoeducational fact sheets (comparator). Materials were delivered remotely in English. Participants in both groups were encouraged to engage with their allocated materials at least weekly during intervention onboarding to promote comparable minimum engagement across conditions, and reminders were provided. The differing delivery formats (self‐directed app vs. online psychoeducational materials) were selected to reflect how these types of supports are typically accessed in real‐world settings. Full details of each condition are provided below.

#### 2.4.1 Intervention Condition: Bud App

Participants allocated to the intervention received access to Bud, a self‐guided digital program designed to support international students' wellbeing. The app targeted modifiable risk factors such as stress, emotion dysregulation, and social isolation, and aimed to strengthen protective factors, including social support, coping skills, and help‐seeking behaviors. Bud incorporated culturally sensitive design elements, including non‐clinical framing, diverse student narratives, and peer‐oriented content.

**FIGURE 1:**
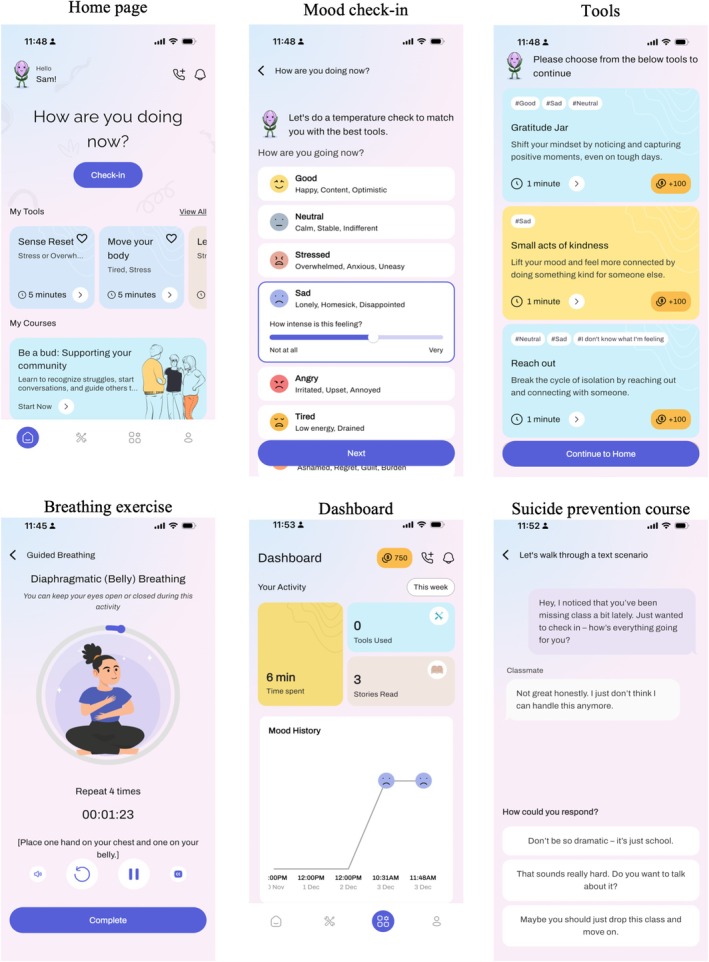
Bud app screenshots.

Key features of Bud were a mood‐tracking feature that recommended contextually relevant tools drawn from acceptance and commitment therapy (ACT), cognitive‐behavioral therapy (CBT), and dialectical behavior therapy (DBT; e.g., cognitive reframing, breathing exercises, temperature‐based distress‐tolerance strategies, structured problem‐solving, values identification); a brief 20‐min suicide‐prevention skills course that provided practical guidance on identifying someone who might be experiencing thoughts of suicide, initiating a supportive conversation, asking directly about suicide, and connecting someone to appropriate professional support; and narrative stories from international students describing their experiences with suicidal thoughts and help‐seeking. The app was not a crisis‐support tool; users were directed to appropriate services when required. Participants engaged with the app flexibly over the four‐week period, consistent with its design as a self‐guided intervention. Participants were encouraged to engage at least weekly and could enable optional daily mood check‐in reminders. This flexible engagement model was intended to reflect real‐world usage patterns of digital mental health tools. Figure 1 below provides example screenshots from the application.

#### 2.4.2 Comparator Condition: Mental Health Fact Sheets

Participants in the comparator group received four brief psychoeducational fact sheets providing mental health information relevant to international students, used in a previous international student mental health study (Carrotte et al. 2026). The fact sheets were representative of resources typically available on university websites and tertiary education platforms, and included stock images to provide a credible low‐intensity intervention rather than a minimal comparator. The fact sheets were developed for research by a registered psychologist with experience in mental health fact sheet writing, but were not co‐designed. Content included maintaining wellbeing through self‐care, building social connections, recognizing when mental health support is needed, and accessing professional services. Fact sheets were delivered through REDCap and were designed to take approximately 5 min each to read (750–1000 words in length). Participants were encouraged to review one fact sheet per week to align with the minimum weekly engagement guidance provided to the intervention group. However, participants were able to access and review the fact sheets flexibly over the four‐week period. Weekly automated reminders were sent via text message to access the scheduled fact sheet for that week if it had not yet been completed.

### 2.5 Assessments

Outcome measures matched those prespecified in the published protocol (McKay et al. 2025). Standardized, validated measures were used for all outcomes, including psychological distress (K10) (Kessler et al. 2002), help‐seeking intentions (GHSQ) (Deane and Wilson 2005), perceived burdensomeness and thwarted belongingness (INQ‐15) (Bryan et al. 2012), emotion dysregulation (DERS‐SF) (Kaufman et al. 2016), suicidal ideation (YSIS‐3) (Hetrick et al. 2021), mental health literacy (MHLS‐9) (Bjørnsen et al. 2024) and User Version of the Mobile Application Rating Scale (uMARS) (Stoyanov et al. 2016). Higher scores reflect greater symptom severity except for help‐seeking and mental health literacy, where higher scores indicate stronger intentions and knowledge, respectively. A summary of the measures and their administration is provided in Table 1. No changes to outcomes or planned analyses occurred after trial commencement. Several measures were completed but not used for this study, including the College Adjustment questionnaire CAQ (O'Donnell et al. 2018) and adverse childhood experiences ACES (Felitti et al. 1998) measures.

**TABLE 1:** Study measures at each time point.

Domain	Measure	# items	T1	T2	T3
Descriptives	Demographics	14	X		
	Previous Suicide attempts	2	X		
	Previous self‐harm behaviors	1	X		
	Adjustment to University: CAQ	14	X		
	Adverse Childhood Experiences: ACEs	11	X		
Primary outcome	Psychological Distress: K10	10	X	X	X
Secondary outcomes	Help‐Seeking: GHSQ	12	X	X	X
	Perceived Burdensomeness: INQ	5	X	X	X
	Sense of Belonging: INQ	5	X	X	X
	Emotion Regulation: DERS – SF	18	X	X	X
	Suicidal ideation: YSIS‐3	3	X	X	X
	Mental health literacy: MHLS‐9	9			X
Acceptability and iatrogenic effects	Open feedback question	1	X	X	X
	Acceptability and Iatrogenic effects: Custom Measure	6			X
	Intervention group only				
	Acceptability of app: uMARS	20			X
	Interview invitation	1			X
Engagement metrics	Comparator group only				
	Number of times fact sheets accessed	Web metrics		X	X
	Intervention group only				
	App usage (number and time accessing)	App metric		X	X
	Number and type of activities completed	App metric		X	X
	Perceive helpfulness of activities	In‐app self‐report		X	X
Total # of survey items per group			I = 96	I = 54	I = 90
			C = 96	C = 54	C = 69

*Note:* Participants in the Bud group completed an additional 21 items at T3 due to the acceptability measures for the app (UMARS) and an interview invitation.

Abbreviations: C, comparator; I, intervention.

### 2.6 Harms

Participant safety was monitored at all assessment time points. Psychological distress (K10) and suicidal ideation (YSIS‐3; both discussed below) were assessed at T1, T2, and T3, and participants were additionally asked whether any study content increased distress or suicidal thoughts. Predefined thresholds (K10 ≥ 30 (Stallman et al. 2010), any suicidal ideation, or distress attributed to study content) triggered an offer of a wellbeing check‐in. Check‐ins were completed by trained team members who conducted a structured phone‐based risk assessment and applied the trial's four‐level risk framework (none, low, moderate, high/life‐threatening). Participants assessed as high/life‐threatening risk were to be withdrawn and referred to emergency services; those at lower risk could continue if safe and willing to do so. Crisis information was provided throughout the study, and all adverse events or iatrogenic responses were recorded.

### 2.7 Sample Size

A priori power analysis, based on 1000 dataset simulations for a Group × Time interaction in a repeated‐measures mixed‐effects model using SIMR in R, indicated that 252 participants (126 per group) would provide approximately 80% power to detect a moderate interaction effect at *α* = 0.05. Allowing for 20% attrition, the planned sample size was 302 participants (151 per group).

### 2.8 Deviations From Protocol

Two minor deviations from the published protocol (McKay et al. 2025) occurred. First, the two funder‐required acceptability items were not administered due to an error in the REDCap branching logic; all other acceptability and iatrogenic‐effects items were delivered as planned. Second, the General Help‐Seeking Questionnaire (GHSQ) was administered using the “emotional problems” reference category rather than the “suicidal ideation” version specified in the protocol, although scoring procedures and analytic treatment were identical. No other deviations from the prespecified outcomes or assessment schedule occurred. The interview data described in the protocol will be reported in a separate manuscript.

### 2.9 Statistical Methods

All analyses followed intention‐to‐treat principles. Primary and secondary outcomes were analyzed using linear mixed‐effects models fitted with the lme4 package in R version 4.5.2, with random intercepts for participants, random slopes for time, and fixed effects for group, time, and their interaction. Type III fixed‐effects tests were obtained using the lmerTest package, and estimated marginal means and contrasts were computed using the emmeans package. Missing outcome data were handled via maximum‐likelihood estimation under a missing‐at‐random assumption. Model diagnostics were used to evaluate distributional assumptions. Because suicidal ideation scores showed excess zeros, alternative count‐model specifications (Poisson, negative binomial, and zero‐inflated Poisson) were fitted as sensitivity analyses; these did not improve model fit or alter conclusions, so results are reported from the primary linear mixed‐effects models. Acceptability and engagement variables were summarized descriptively, and correlations between engagement and mental‐health outcomes were examined. Exploratory moderation analyses tested whether demographic factors or baseline suicidal‐ideation strata influenced intervention effects, and sensitivity analyses assessed whether outcome patterns varied by Bud app engagement. All hypothesis tests were two‐tailed with *α* = 0.05.

### 2.10 Patient and Public Involvement

The Bud app was developed through an extensive co‐design process with international students and sector stakeholders (Ng 2026). The expert advisory group comprised five international students and two sector representatives, who contributed to identifying priorities, refining the intervention concept, and shaping content and interface design. International students participated in 10 iterative workshops that informed the focus, content, wording, cultural framing, safety features, and delivery format. Students were compensated for their time. No members of the public were involved in data collection, analysis, or interpretation beyond these co‐design roles, and they did not contribute to authorship.

## 3 Results

### 3.1 Demographics and Baseline Characteristics

**FIGURE 2:**
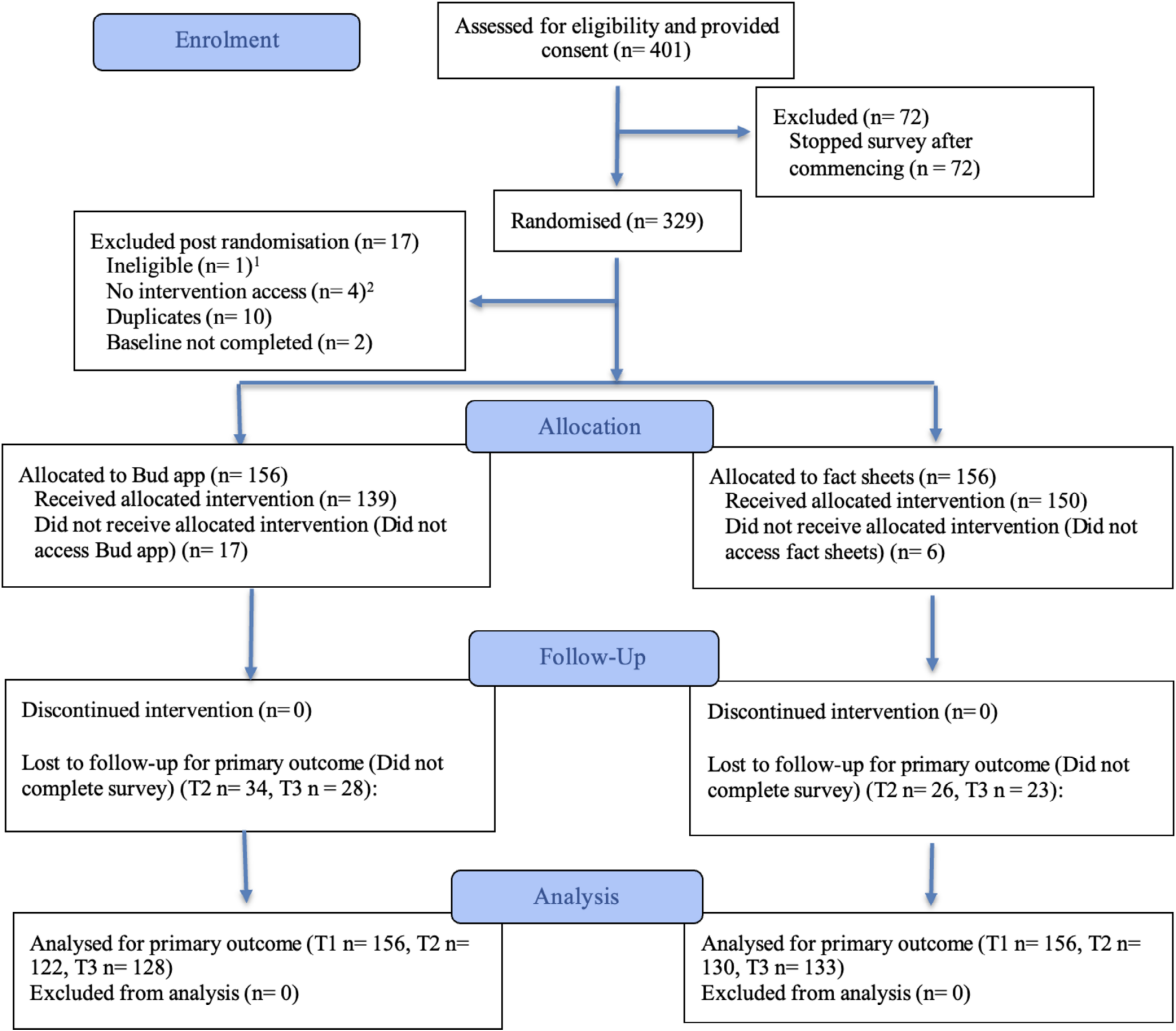
Consort flow diagram. 1 = Participant met eligibility criteria at start of study but returned to home country and was therefore no longer eligible. 2 = Participants did not receive links to access interventions due to a technical issue with REDCap.

Of the 401 participants who passed eligibility checks and provided consent, 329 progressed far enough through the baseline survey to be automatically randomized. Seventeen participants were excluded after randomization (see Figure 2), leaving a final sample of 312, with 156 allocated to the Bud group and 156 to the comparator group. Baseline characteristics were comparable across groups, with no meaningful imbalances in demographic or clinical variables (See Tables 2 and 3).

**TABLE 2:** Sample demographics.

Variable	Full sample	Bud	Comparator
Age	25.2 (4.6)	25.1 (4.9)	25.4 (4.4)
Age arrived in Australia	23.0 (5.2)	22.9 (5.5)	23.0 (4.8)
Self‐rated English ability out of 10	8.1 (1.5)	8.1 (1.5)	8.1 (1.4)
Gender			
Female	213 (68.3%)	101 (64.7%)	112 (71.8%)
Male	91 (29.2%)	50 (32.1%)	41 (26.3%)
Non‐binary	5 (1.6%)	3 (1.9%)	2 (1.3%)
Prefer not to answer	2 (0.6%)	2 (1.3%)	0 (0.0%)
Different term	1 (0.3%)	0 (0.0%)	1 (0.6%)
Education level			
Postgraduate	187 (59.9%)	92 (59.0%)	95 (60.9%)
Undergraduate	108 (34.6%)	59 (37.8%)	49 (31.4%)
Certificate/Diploma	11 (3.5%)	4 (2.6%)	7 (4.5%)
Other	6 (1.9%)	1 (0.6%)	5 (3.2%)
Top 3 Countries of birth^a^			
China	86 (27.6%)	44 (28.2%)	42 (26.9%)
India	49 (15.7%)	26 (16.7%)	23 (14.7%)
Indonesia	41 (13.1%)	17 (10.9%)	24 (15.4%)
Past 12 Months Suicidal Thoughts and Behaviors			
Seriously considered suicide	46 (14.7%)	21 (13.5%)	25 (16.0%)
Made a suicide attempt	4 (1.3%)	1 (0.6%)	3 (1.9%)
Self‐harmed	45 (14.4%)	21 (13.5%)	24 (15.4%)

*Note: N* = 312.

^a^ There was a total of 40 countries represented in the sample.

**TABLE 3:** Baseline scores for main variables.

Variable	Full sample		Bud		Comparator	
	M	SD	M	SD	M	SD
Psychological distress (K10)	22.61	7.33	22.33	7.80	22.89	6.84
Help seeking intentions (GHSQ)	35.96	9.95	35.65	10.54	36.26	9.35
Perceived burdensomeness (INQ)	9.12	5.70	8.86	5.34	9.37	6.06
Thwarted belongingness (INQ)	17.88	6.09	17.50	6.39	18.26	5.77
Emotion regulation difficulties (DERS‐SF)	45.66	12.48	44.28	12.11	47.04	12.72
Suicidal ideation (YSIS‐3)	0.78	1.64	0.71	1.65	0.85	1.63
Suicidal ideation (SI > 0 only)	3.38	1.69	3.58	1.84	3.22	1.57

*Note: N* = 312. Suicidal ideation (SI > 0 only) reflects the mean among participants reporting any suicidal ideation at baseline and aligns with best practice reporting standards (McKay and Spittal 2025). Overall, 72 participants (23.1%) reported suicidal ideation (score > 0), with similar proportions across groups (Bud: 31 participants, 19.9%; Fact sheets: 41 participants, 26.3%).

### 3.2 Internal Pilot Feasibility Phase

All internal pilot thresholds were met, with participant recruitment completed within 3 months (green = > 50% recruitment within 6 months), 80.1% of participants completing follow‐up surveys (green = > 60% completing follow‐up surveys) and 71.2% of users engaging with the app at least three times (green = ≥ 50% using the app at least three times). No primary or secondary outcome data were examined during this phase, and all data collected were retained for analysis.

### 3.3 Effectiveness

For the primary outcome of psychological distress (H1), both groups showed reductions from baseline (T1) to post‐intervention (T3). However, the hypothesis was not supported as the patterns of change were similar across the Bud and Fact sheet groups, and there was no evidence of differential improvement between conditions (See Table 4). Across secondary efficacy outcomes, the hypotheses were also not supported, as the estimated marginal means followed comparable trajectories in the two groups. Help‐seeking intentions (H2), perceived burdensomeness, and thwarted belongingness (H3) remained relatively stable over time, while emotion dysregulation (H4) and suicidal ideation decreased (H6) in both groups. No Group × Time interactions were detected for any of these outcomes, and all associated effect sizes were small. Similarly, at the final time point, self‐reported changes in mental health literacy from the interventions did not differ between groups, *t*(252.2) = 0.85, *p* = 0.396 (Bud: M = 27.50, SD = 7.13; Fact sheets: M = 26.76, SD = 6.75), not supporting H5.

**TABLE 4:** Model‐estimated outcomes over time by intervention group.

Outcome	Group	Estimated marginal means			Time effect	Group × Time	Cohen's *D*
		Time 1	Time 2	Time 3	F (DF) P	F (DF) P	Interaction
*Primary outcome*							
Psychological Distress	I	22.33 (0.59)	21.47 (0.60)	20.10 (0.62)	**19.49 (2, 342.45), *p* < 0.001**	0.43 (2, 342.45), *p* = 0.652	−0.09 [−0.32, 0.15]
	C	22.89 (0.59)	21.59 (0.60)	20.74 (0.61)			
*Secondary outcomes*							
Help Seeking Intentions	I	35.65 (0.78)	35.85 (0.86)	36.40 (0.87)	0.69 (2, 515.61), *p* = 0.502	0.45 (2, 515.61), *p* = 0.636	0.02 [−0.22, 0.26]
	C	36.26 (0.78)	35.60 (0.85)	36.24 (0.86)			
Perceived Burdensomeness	I	8.86 (0.45)	8.60 (0.46)	8.34 (0.44)	1.99 (2, 525.00), *p* = 0.138	0.05 (2, 525.00), *p* = 0.948	−0.11 [−0.32, 0.11]
	C	9.37 (0.45)	9.27 (0.46)	8.96 (0.44)			
Thwarted Belongingness	I	17.50 (0.49)	17.89 (0.50)	17.51 (0.50)	0.79 (2, 357.97), *p* = 0.453	0.28 (2, 357.97), *p* = 0.758	−0.08 [−0.31, 0.15]
	C	18.26 (0.49)	18.27 (0.49)	18.01 (0.50)			
Emotion regulation difficulties	I	44.28 (0.98)	42.87 (1.01)	41.38 (1.02)	**24.30 (2, 365.13), *p* < 0.001**	2.37 (2, 365.13), *p* = 0.095	−0.09 [−0.31, 0.14]
	C	47.04 (0.98)	43.46 (1.00)	42.48 (1.01)			
Suicidal Ideation	I	0.71 (0.13)	0.52 (0.12)	0.37 (0.11)	**5.35 (2, 347.32), *p* = 0.005**	0.42 (2, 347.32), *p* = 0.660	−0.14 [−0.34, 0.05]
	C	0.85 (0.13)	0.59 (0.12)	0.60 (0.11)			

*Note:* 95% confidence intervals for Cohen's *D* are shown in square brackets.

Abbreviations: C, comparator; I, Bud group.

Sensitivity analyses testing the impact of engagement with the Bud app (median split) showed no differences in outcomes, and there was no significant correlation between engagement and mental health outcomes at the end of the study. Suicidal ideation was also analyzed using zero‐inflated Poisson models to account for excess zeros; these sensitivity analyses yielded the same pattern of results, with no evidence of differential change between groups. Exploratory moderation analyses stratified by baseline suicidal ideation yielded the same conclusions for the primary outcome and all but two secondary outcomes, and small interactions for perceived burdensomeness and suicidal ideation did not alter the overall interpretation of the trial. Additional exploratory moderation analyses examining gender (male vs. female) showed no meaningful moderation of change over time for any outcome; a small interaction for perceived burdensomeness reflected only trivial, non‐divergent fluctuations and did not influence the overall conclusions.

### 3.4 Acceptability

In the Bud group, 99.2% rated the intervention as at least somewhat enjoyable, and 95.2% rated it as worthwhile. Most participants (88.9%) reported that it was not at all upsetting, with 11.1% describing the app as somewhat upsetting. At T3, participants generally provided positive ratings across acceptability domains, including functionality (M = 4.07, SD = 0.53), esthetic appeal (M = 3.97, SD = 0.65), information quality (M = 4.05, SD = 0.58), and perceived health impact (M = 3.74, SD = 0.74), with more neutral ratings for engagement (M = 3.40, SD = 0.60) and overall quality (M = 3.67, SD = 0.88).

#### 3.4.1 Engagement

Eighty‐nine percent (*n* = 139) of participants assigned to the Bud group downloaded and used the app. Participants completed a mean of 17.99 (SD = 20.76; median = 10) activities (e.g., mood check‐ins, tools, stories, or course modules). Engagement was highest early in the intervention period, although some participants did not start using the app immediately. In week 1, 125 participants (80.1% of those allocated) recorded at least one engagement event. This declined to 91 participants (58.3%) in week 2 and 83 participants (53.2%) in week 3. Engagement then stabilized, with 84 participants (53.8%) recording at least one engagement in the period up to their final survey.

In terms of specific content, mood check‐ins were used by all participants who accessed the app (100%, *n* = 139), with a median of 5 check‐ins recorded across the 4‐week access period. Tool engagement was also high, with 123 participants (88.5% of users) accessing at least one tool, with a median of four tools completed. Story engagement was modest: 25.9% (*n* = 36) viewed at least one story (median = 4.5). Engagement with the suicide‐prevention skills course for supporting others was lower: 17.3% (*n* = 27) began the course, and 9.6% (*n* = 15) completed it, indicating that 55.6% of those who started went on to complete the full course. Of 370 in‐app helpfulness ratings, 95.4% were “helpful,” with similar patterns across tool types (see Table S1). In the comparator group, 150 participants (96.2%) accessed the fact sheets, and most read all four (*n* = 135, 90.0%; M = 3.69, SD = 0.90).

### 3.5 Iatrogenic Effects

Iatrogenic responses were rare. Thirteen participants requested at least one safety callback during the study, with between one and three callbacks per person, and a total of 18 callbacks were completed. Of these, 11 were assessed as involving no suicide risk, and seven were assessed as involving low suicide risk based on the study's predefined criteria. At the end of the trial, no participants in the Bud group and one participant in the fact sheets group (0.8%) indicated that the materials had made them feel suicidal, unsafe, or prompted an urge to self‐harm, and no participants in either group rated the materials as very upsetting.

## 4 Discussion

This trial aimed to evaluate the effectiveness, acceptability, engagement, and safety of Bud, a co‐designed digital intervention targeting upstream suicide‐related risk and protective factors among international students. Across outcomes, participants assigned to Bud and the active psychoeducational comparator showed reductions in distress, emotion dysregulation, and suicidal ideation over the four‐week period. These improvements were comparable across groups. However, none of the primary (H1) or secondary ([Link], [Link], [Link], [Link], [Link]) effectiveness hypotheses predicting greater changes in the Bud group were supported. In contrast, supporting the acceptability, engagement, and safety hypotheses ([Link], [Link], [Link]), Bud was rated as acceptable, safe, and worthwhile, and app uptake and activity completion were moderate to high. Taken together, the findings indicate that Bud is feasible and acceptable to international students but was not superior to a psychoeducational comparator within the brief trial window.

Several factors likely contributed to the absence of group differences. First, the comparator was active, adherence to the fact sheets was very high, and their structured content functioned as a low‐intensity wellbeing intervention rather than a minimal control. Providing reliable, clear mental health information is likely beneficial for international students who face barriers to help‐seeking and report low mental health literacy (Clough et al. 2019; Sanci et al. 2022), reducing the potential for between‐group contrasts. Second, the four‐week dose may have been insufficient for shifting transdiagnostic mechanisms such as perceived burdensomeness and belongingness. These constructs typically change gradually, often requiring sustained practice or therapist‐supported work, and effects are strongest in clinical rather than community samples (Bianchini and Bodell 2024; Hill and Pettit 2019; Moltrecht et al. 2021). Third, baseline distress and suicidal ideation were moderate, creating a floor effect that limited the scope for larger symptom reductions. Finally, non‐specific influences such as repeated assessment, exposure to supportive material, normalization of distress, and regression to the mean likely contributed to parallel improvements in both arms (Mohr et al. 2009; Morton and Torgerson 2005). Although Bud targeted proximal factors implicated in contemporary suicide theories, such as interpersonal risk processes (Van Orden et al. 2010; Robinson et al. 2018), these mechanisms may require more sustained or structured intervention dosing than a brief self‐guided program can deliver in 4 weeks.

These explanations are consistent with broader digital mental health research. Across university populations, digital interventions frequently outperform waitlist or no‐control conditions but show smaller differences compared with active comparators (e.g., treatment‐as‐usual referrals, matched attention conditions) (Madrid‐Cagigal et al. 2025; Farrer et al. 2024; Stallman et al. 2019). Viewed within this wider evidence base, the parallel improvements across groups align with existing findings that light‐touch digital supports and credible psychoeducational content each produce short‐term reductions in distress and suicidal ideation (Madrid‐Cagigal et al. 2025; Torok et al. 2022). Evidence also indicates that guided or partially supported digital interventions, even with non‐clinicians, often produce larger effects than fully self‐guided formats (Leung et al. 2022; Plessen et al. 2025), and this may further explain the limited changes in the current trial. The findings suggest that mechanisms which prompt engagement with mental health content designed specifically for international students may be particularly important. Although much of the underlying information was already available through public and institutional websites, outcomes still improved when students were prompted to engage with content that was tailored, contextualized, and framed for their experiences, indicating that relevance and delivery, not access alone, may drive benefit. Beyond these outcomes, patterns of acceptability and engagement provide insight into how international students interact with digital suicide‐prevention content.

Almost all Bud users rated the app as enjoyable and worthwhile, with strong evaluations of functionality, esthetics, and information quality. Engagement was highest for short, actionable components such as emotion check‐ins and brief tools. Uptake of narrative stories and the suicide‐prevention skills course was lower, but they were also less central to the primary user flows (e.g., emotion check‐in followed by recommended tools), which may explain their lower usage. Although engagement did not predict outcomes, the patterns generally suggest that students prefer concise, immediately accessible, and helpful strategies. Engagement levels were broadly consistent with findings from the MindYourHead study among Chinese international students in Australia, where most users who accessed the intervention completed at least one module and many completed multiple modules (Choi et al. 2023). Together, these studies indicate that international students engage reliably with culturally relevant, self‐paced digital content, though the MindYourHead findings also highlight that native‐language options may support greater uptake in future iterations of Bud.

Feasibility and safety outcomes were positive. Recruitment proceeded efficiently, retention exceeded the prespecified threshold, and most Bud users engaged with the app multiple times. Safety protocols operated as intended, with few callbacks required, no high‐risk cases detected, and one participant in the factsheet group and no participants in the bud group reporting the intervention increased suicidal thoughts or distress. The trial demonstrates that an RCT evaluating a suicide‐prevention app with explicit measurement of suicidal ideation and structured risk monitoring can be undertaken safely with international students. More broadly, the results indicate that international students respond well to structured mental health information delivered in culturally attuned, low‐intensity digital formats. Clear guidance, normalization of distress, and simple self‐management options can be provided safely at scale and may represent an acceptable entry point to mental health information and intervention (McKay and Meza 2024; Clough et al. 2019; Sanci et al. 2022).

The findings have several implications for research and practice. First, low‐intensity, culturally responsive digital supports are a viable and acceptable component of a stepped approach for international students, supporting broader calls for such approaches in higher education settings (Brennenstuhl et al. 2025). Second, psychoeducation remains a scalable option that can produce meaningful short‐term improvements when delivered in a structured format that encourages engagement. Third, future versions of Bud may require stronger targeting of key mechanisms, more sustained exposure, or closer alignment between content and hypothesized targets such as belongingness. These refinements should be coupled with implementation research to determine how universities can integrate digital interventions into existing wellbeing systems, including timing of delivery during periods of elevated stress, such as when transitioning to the new environment (Aljaberi et al. 2021).

Strengths of the study include being the first randomized evaluation of a co‐designed suicide‐prevention app for international students, an adequately powered sample with stratified randomization, rigorous mixed‐effects and zero‐inflated modeling, and the use of prespecified safety procedures. Limitations include the short duration of follow‐up, reliance on self‐report measures, and having the interventions only in English. In addition, while the fact sheet condition aligned with typical existing supports, it was not a mental health app, reducing comparability between conditions in both student engagement and the types of activities completed. We also did not include a non‐active control condition, which limits our ability to determine whether changes in outcomes were attributable to the interventions rather than to non‐intervention effects such as regression to the mean.

Future work should examine Bud over longer periods and track whether effects consolidate or diverge beyond the initial four‐week window. Including a non‐active comparator (e.g., waitlist control) or alternative app‐comparator conditions would also strengthen potential conclusions regarding efficacy. Similarly, testing the intervention in clinical populations, with more distressed subgroups, or as an adjunct to institutional services may clarify the contexts in which added benefit emerges. Refinements should focus on increasing the salience and uptake of mechanism‐focused app components, potentially through adaptive delivery or micro‐randomized designs that identify which tools are most effective for which students at which moments. Finally, implementation studies are needed to determine how to embed Bud within university systems and assess its cost‐effectiveness at scale.

## 5 Conclusion

Bud was feasible, acceptable, and safe, and both intervention arms showed meaningful short‐term improvements. Although Bud did not demonstrate superiority to a structured psychoeducational comparator, the trial provides foundational evidence for the safe delivery of a co‐designed, suicide prevention‐focused digital intervention for international students. Longer‐term, mechanism‐focused, and implementation‐oriented research is needed to determine how such tools can most effectively support culturally diverse students in reducing distress and suicide risk.

## Author Contributions

**Samuel McKay:** conceptualization, methodology, formal analysis, writing – original draft, writing – review and editing, supervision, resources, project administration, funding acquisition. **Michelle Lamblin:** conceptualization, funding acquisition, writing – review and editing. **Jennifer Nicholas:** conceptualization, methodology, funding acquisition, writing – review and editing. **Vivienne Browne:** conceptualization, methodology, funding acquisition, writing – review and editing. **Gina Chinnery:** conceptualization, methodology, funding acquisition, writing – review and editing. **Christina Ng:** methodology, project administration, writing – review and editing. **Gregory Armstrong:** conceptualization, funding acquisition, writing – review and editing. **Ellie Brown:** supervision, writing – review and editing. **Elise Carrotte:** methodology, resources, writing – review and editing. **Madhavan Mani:** software, writing – review and editing. **Jo Robinson:** conceptualization, supervision, funding acquisition, resources, writing – review and editing. **Isabella Choi:** conceptualization, methodology, funding acquisition, writing – review and editing. **Bailey Nation‐Ingle:** conceptualization, methodology, writing – review and editing. **Kristal Allison:** conceptualization, writing – review and editing. **Ella Perlow:** conceptualization, writing – review and editing. **Jocelyn I. Meza:** conceptualization, writing – review and editing, funding acquisition.

## Funding

This work was funded by a Study Melbourne Inclusion Program Grant from the Victorian Government Department of Jobs, Skills, Industry and Regions, An Early Career Researcher Grant from the University of Melbourne, and an Innovation Grant from Suicide Prevention Australia Limited. Jo Robinson is funded by a National Health and Medical Research Council Investigator Grant (ID2008460) and University of Melbourne: DameKateCampbellFellowship. Ellie Brown is funded by the University of Melbourne: RonaldPhilipGriffithFellowship.

## Conflicts of Interest

Dr. Mani was contracted and paid to develop the Bud app evaluated in this study. The contract was completed prior to trial commencement, and Dr. Mani had no role in outcome selection, data analysis, or interpretation. The remaining authors declare no conflicts of interest.

## Supporting information


**Table S1:** Bud Tool engagement and ratings of helpfulness

**File S2:** CONSORT 2025 Checklist.

## Data Availability

The data that support the findings of this study are available on request from the corresponding author. The data are not publicly available due to privacy or ethical restrictions.
