# Supplementary material for: A Randomized Controlled Trial With an Internal Pilot of a Co‐Designed App Targeting Upstream Suicide‐Related Risk and Protective Factors Among International Students
Source: Suicide Life Threat Behav. 2026 Jul 2;56(4):e70125. doi: 10.1111/sltb.70125 (PMC13324967; doi:10.1111/sltb.70125)
Supplement: Supplementary file 1 — Table S1: Bud Tool engagement and ratings of helpfulness [file SLTB-56-0-s001.docx]

**Supplementary Table 1.**

*Bud Tool engagement and ratings of helpfulness*

| **Tool Name** | **Total Access Count** | **No. of Users Accessed** | **Total helpful** | **Total Unhelpful** |
| --- | --- | --- | --- | --- |
| Celebrate your happiness | 213 | 65 | 52 | 0 |
| Sense Reset | 194 | 75 | 43 | 1 |
| Gratitude Jar | 146 | 70 | 48 | 1 |
| Move your body | 127 | 52 | 27 | 2 |
| Let it go | 101 | 49 | 35 | 1 |
| Reach out | 87 | 49 | 23 | 2 |
| Weekly bucket list | 53 | 39 | 24 | 0 |
| Take a Breather | 46 | 29 | 17 | 3 |
| Worry Jar | 40 | 30 | 10 | 1 |
| Write it out | 38 | 28 | 11 | 1 |
| Kind Touch | 33 | 20 | 9 | 0 |
| Name the feeling | 23 | 21 | 6 | 0 |
| Temperature change | 23 | 16 | 7 | 0 |
| What’s in my control | 18 | 17 | 5 | 0 |
| Find Your Why | 14 | 13 | 6 | 1 |
| Guided Breathing | 13 | 10 | 4 | 1 |
| The future looks bright | 13 | 12 | 5 | 0 |
| A bit more support | 12 | 9 | 5 | 0 |
| The Reset | 11 | 11 | 1 | 0 |
| Small acts of kindness | 10 | 9 | 1 | 1 |
| Redirect your energy | 10 | 8 | 3 | 1 |
| If it were a friend | 10 | 10 | 3 | 1 |
| The problem solver | 9 | 8 | 2 | 0 |
| Finding Friends | 9 | 6 | 0 | 0 |
| Frameshift | 6 | 5 | 3 | 0 |
| Your Value Reflection | 4 | 4 | 3 | 0 |
